# Hypersensitivity reaction to intravenous ondansetron: A case report

**DOI:** 10.1002/ccr3.6363

**Published:** 2022-09-24

**Authors:** Sagar Devkota, Umesh Pradhan, Ishu Gajurel

**Affiliations:** ^1^ Anesthesiology Sindhuli Hospital Kathmandu Nepal; ^2^ General Surgery Sindhuli Hospital Kathmandu Nepal; ^3^ GP and Emergency Medicine Sindhuli Hospital Kathmandu Nepal

**Keywords:** Hypersensitivity reaction, Ondansetron

## Abstract

Ondansteron is widely used as prophylaxis for postoperative nausea and vomiting and is associated with various side effects. We present a case of hypersensitivity reaction in the form of itching and rashes in a 6‐year‐old female patient with a single dose of intravenous ondansetron.

## Abstract 2

Even though hypersensitivity reactions associated with ondansetron are rare, we should all be cautious while administering the drug and should be prepared with all necessary emergency drugs and equipment.

## 1 INTRODUCTION

Ondansetron hydrochloride, a 5‐Hydroxytryptamine (5‐HT3) receptor antagonist, is a widely used antiemetic agent used for nausea and vomiting related to chemotherapy and as a prophylactic agent for postoperative nausea and vomiting (PONV).^1^ It is known to be associated with various adverse reactions like headache, constipation, or diarrhea. There are case reports showing cardiac arrest following rapid intravenous push of ondansetron.^2^ We present a 6‐year‐old female patient planned for incision and drainage under intravenous anesthesia that developed an episode of localized itching and rash due to intravenous dose of ondansetron.

## 2 CASE PRESENTATION

A 6‐year‐old female patient was brought to surgery outpatient department with swelling of right hand for 1 week. She was diagnosed with right‐hand abscess and was planned for incision and drainage under intravenous anesthesia. She had no history of any previous allergic manifestations or reactive airway diseases. She was taken to the operating room and incision and drainage was performed. After she was fully awake, she complained of nausea. Immediately after giving ondansetron (2 mg IV), she developed localized itching and had rashes over the left forearm. Her vitals were stable, and she did not complain of any respiratory distress or dizziness. She was given hydrocortisone (100 mg IV) and chlorpheniramine maleate (5 mg slow IV) after which the rashes and itching subsided. She was kept under observation for 24 h and remained asymptomatic. She was discharged on the second postoperative day. On retrospective evaluation of the patient, her parents denied any history of allergic reactions to any food or medications.

## 3 DISCUSSION

Intravenous anesthetics drugs like fentanyl and ketamine are associated with postoperative nausea and vomiting, which is a very common and distressing complication following surgery.^3^ The average incidence of PONV with a single risk factor is 10% with risks as high as 80% as the risk factors increase.^4^ Usually with a single risk factor, no or a single antiemetic agent‐like 5HT3 receptor antagonist is given. Ondansetron, a highly selective 5HT3 receptor antagonist, is very effective as a prophylactic agent for PONV. Most common side effects^2^ associated with the use of this drug are headache, constipation or diarrhea and stomachache, a muscle spasm or temporary loss of vision being the rarer side effects.

Hypersensitivity reactions characterized by rashes, itching, edema gradually progressing to shortness of breath and sudden cardiac arrest have been reported in patients who had a history of such allergic reactions to other selective 5HT3 receptor antagonists.^5^ However, such severe reaction without prior history and other predisposing risk factors is rare. These reactions are labeled by some authors as class effect and drug‐specific reaction by some. On the Naranjo's casualty assessment scale, this adverse reaction was 6, which indicated a probable reaction to the drug.^6^

Even though a rare presentation, anesthesiologists should always consider the possibility of these hypersensitivity reactions during preanesthetic evaluation, and however minor the operative procedure may be, one should always be prepared with all the emergency drugs and equipment.

## 4 CONCLUSION

Despite the lack of enough resources to properly describe this reaction is due to ondansetron, it can be either anaphylaxis or anaphylactoid reaction with the latter being a more likely cause as there was no previous exposure to this drug. We should all be cautious while administering this drug and should be prepared with all the necessary emergency equipment and drugs.

## AUTHOR CONTRIBUTIONS

SD conceptualized the study, was in charge of the case, wrote the original manuscript, and reviewed and edited the manuscript. UP and IG reviewed and edited the manuscript.

## ETHICAL APPROVAL

Written informed consent was obtained from the parents as the patient was a minor, for the publication of the case report.

## CONSENT

Written informed consent was obtained from the parents.
